# Outdoor malaria vector species profile in dryland ecosystems of Kenya

**DOI:** 10.1038/s41598-022-11333-2

**Published:** 2022-05-03

**Authors:** Fiona Kinya, Clifford M. Mutero, Rosemary Sang, Eunice A. Owino, Gilbert Rotich, Edwin O. Ogola, Charles S. Wondji, Baldwyn Torto, David P. Tchouassi

**Affiliations:** 1 International Centre of Insect Physiology and Ecology, P.O. Box 30772-00100, Nairobi, Kenya; 2 University of Nairobi, P.O. Box 30197-30100, Nairobi, Kenya; 3 School of Health Systems and Public Health, University of Pretoria, Private Bag X323, Pretoria, 0001 South Africa; 4 Department of Vector Biology, Liverpool School of Tropical Medicine, Pembroke Place, Liverpool, L35QA UK; 5 LSTM Research Unit at the Centre for Research in Infectious Diseases (CRID), P.O. Box 1359, Yaoundé, Cameroon; 6 Department of Zoology and Entomology, University of Pretoria, Private Bag X323, Pretoria, 0001 South Africa

**Keywords:** Ecology, Malaria

## Abstract

Outdoor biting by anopheline mosquitoes is one of the contributors to residual malaria transmission, but the profile of vectors driving this phenomenon is not well understood. Here, we studied the bionomics and genetically characterized populations of *An. gambiae* and *An. funestus* complexes trapped outdoors in three selected dryland areas including Kerio Valley, Nguruman and Rabai in Kenya. We observed a higher abundance of *Anopheles funestus* group members (n = 639, 90.6%) compared to those of the *An. gambiae* complex (n = 66, 9.4%) with *An. longipalpis* C as the dominant vector species with a *Plasmodium falciparum* sporozoite rate (*Pf*sp) of 5.2% (19/362). The known malaria vectors including *An. funestus* s.s. (8.7%, 2/23), *An. gambiae* (14.3%, 2/14), *An. rivulorum* (14.1%, 9/64), *An. arabiensis* (1.9%, 1/52) occurred in low densities and displayed high *Pf*sp rates, which varied with the site. Additionally, six cryptic species found associated with the *An. funestus* group harbored *Pf* sporozoites (cumulative *Pf*sp rate = 7.2%, 13/181). We detected low frequency of resistant 119F-*GSTe2* alleles in *An. funestus* s.s. (15.6%) and *An. longipalpis* C (3.1%) in Kerio Valley only. Evidence of outdoor activity, emergence of novel and divergent vectors and detection of mutations conferring metabolic resistance to pyrethroid/DDT could contribute to residual malaria transmission posing a threat to effective malaria control.

## Introduction

Malaria case incidence and mortality have fallen in Kenya by 58% and 11%, respectively, since the early 2000s consistent with the global trend^1–3^. This reduction has been achieved owing to combined interventions of long-lasting insecticidal nets (LLINs), indoor residual spraying (IRS), prompt diagnosis and treatment with artemisinin-based combination therapies and intermittent preventive treatment of pregnant women^2^. Despite this progress, the disease still exerts a huge health burden accounting for an estimated 19% of all outpatient consultations in Kenya^4^. This trend is potentially indicative of the disease persistence and facilitated by several factors including house characteristics, use of bednets or other control measures, human activities and behavior, and vector-related factors^5^. However, entomologic determinants remain critical. For instance, resistance to insecticides has developed and spread among major malaria vectors^6^. Also noted are changes in *Anopheles* vector composition following sustained vector control, as well as resting and biting habits especially outdoors^7^. Collectively, these factors limit the effectiveness of LLINs and IRS mainly deployed indoors resulting in residual malaria transmission (RMT) which is defined as ongoing transmission despite 100% implementation of LLINs and IRS fully susceptible to local vectors^7,8^

Outdoor biting has received increasing attention regarding control of malaria towards elimination. A recent modeling study predicted that across Africa, outdoor transmission resulted in an estimated 11 million additional malaria cases annually assuming universal LLIN and IRS coverage^8^. Thus, the contribution of outdoor biting to malaria persistence could be much higher as universal coverage of ITNs and IRS has not been achieved. A multicountry study in Cameroon, Kenya and Ethiopia found high malaria transmission outdoors compared to indoors^5^. While this phenomenon is not new^9–11^, the profile of vectors implicated in outdoor transmission could differ in different eco-epidemiologic settings^5^.

In Kenya, monitoring efforts have assessed the malaria epidemiologic landscape and identified the key vector species. Primary malaria vectors belong to *Anopheles gambiae* sensu lato (s.l.) and *Anopheles funestus* group, like most of East Africa^9,12,13^. The sibling species in these complexes are morphologically similar as adults, although they have distinct ecological preferences, vectorial capacity and behavior, which impact differentially on malaria epidemiology and control efforts^14,15^. Most comprehensive data on entomologic risk come from the high malaria endemic Lake and Coastal zones. Further, studies in these foci have implicated previously unrecognized species in these complexes in malaria transmission^16,17^. This trend has been facilitated by application of sensitive molecular techniques including PCR and sequencing in entomologic surveillance and becoming increasingly crucial to identify new threats, availing opportunities for directing interventions^12,16–18^.

The present study was designed to gain insights into malaria persistence in dryland ecosystems, where malaria is thought to be seasonal and prone to epidemics^4^. Specifically, we focused on outdoor transmission, and describe the species composition in the *An. gambiae* s.l. and *An. funestus* group and natural infection rates with *Plasmodium falciparum* supported by molecular analyses. To determine if the high prevalence of *An. funestus* group members was due to insecticide resistance, we screened them for the presence of mutations in the glutathione S-transferase epsilon 2 gene (*GSTe2*) that confer resistance to pyrethroid-based insecticides and dichloro-diphenyl-trichloroethane (DDT)^19^. The findings from this study have implications for guiding control of RMT towards elimination.

## Results

### *Anopheles funestus* mosquitoes dominate outdoor anopheline catches

**Figure 1:**
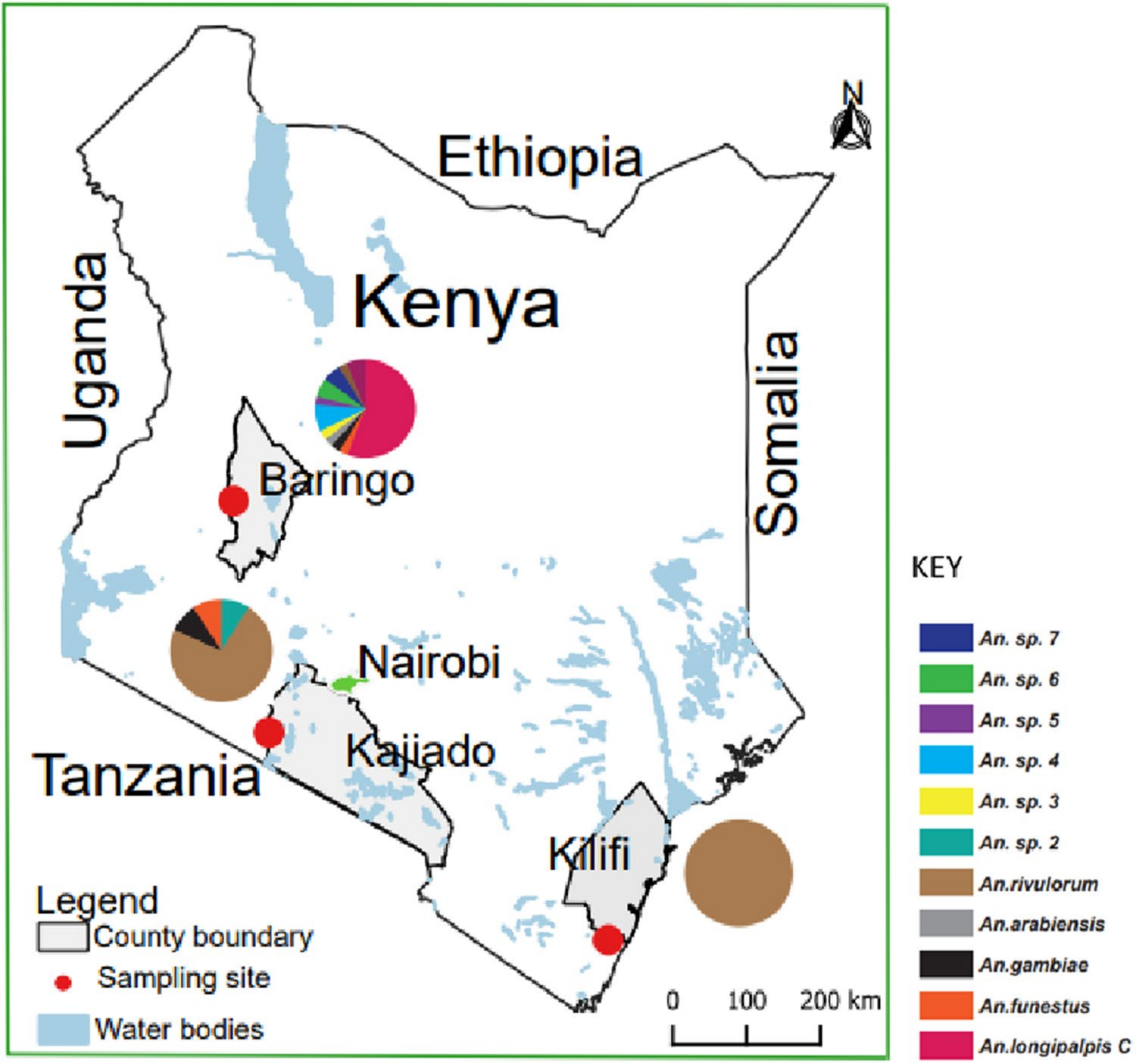
Map of Kenya showing the study areas and distribution of anopheline species found infected with *Plasmodium falciparum* sporozoites. Pie-chart indicating the relative abundance of the species infected based on actual numbers is shown for each study area. The map was designed using ArcMap 10.2.2 with the ocean and lakes base layer derived from Natural Earth (http://www.naturalearthdata.com/, a free GIS data source). The sample points were collected using a GPS gadget (garmin etrex 20, https://buy.garmin.com/en-US/US/p/518046), and the county boundaries for Kenya derived from Africa Open data (https://africaopendata.org/dataset/kenya-counties-shapefile, license Creative Commons).

**Figure 2:**
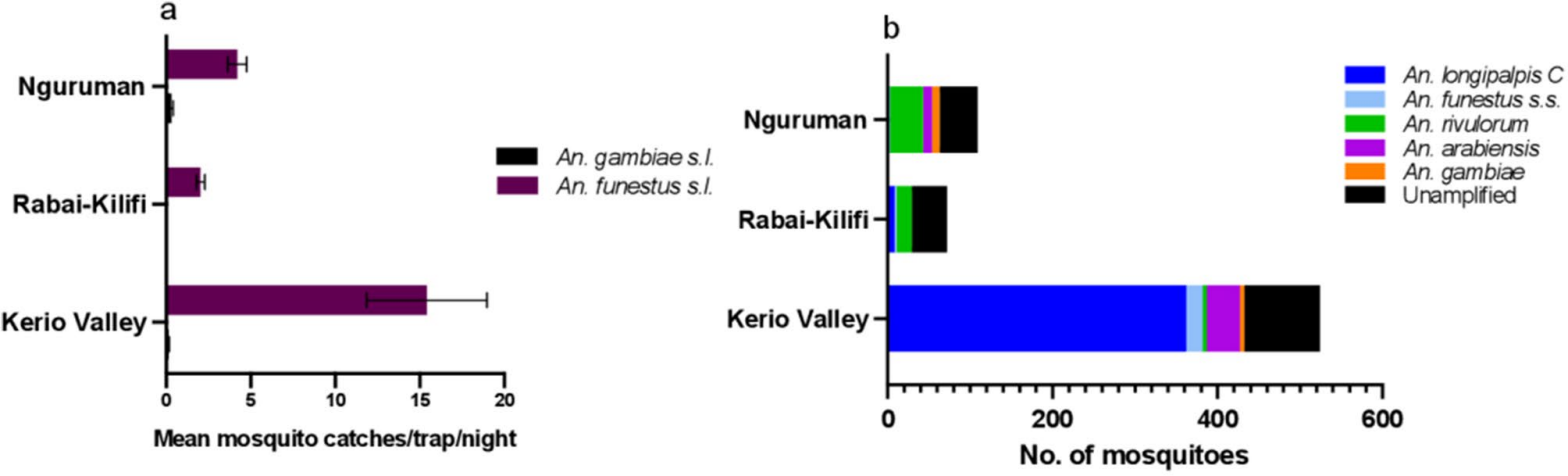
(**a**) Outdoor mean catches/trap/day, (**b**) Species composition, in three study areas in dryland areas of Kenya. The number of trap nights were 44, 50 and 56 in Kerio Valley, Rabai and Nguruman, respectively.

Of the mosquitoes morphologically identified, *An. funestus* s.l. was predominant across the three study areas (Kerio Valley, Nguruman, Rabai; Fig. 1) comprising 90.6% (639/705) of the total captures. Only 9.4% (66/705) were *An. gambiae* s.l. The breakdown of the catches by study areas were as follows: Kerio Valley (*An. funestus* s.l. = 478, *An. gambiae* s.l*.* = 46); Rabai (*An*. *funestus* s.l. = 72, *An. gambiae* s.l. = 0) and Nguruman (*An. funestus* s.l*.* = 89, *An. gambiae* s.l. = 20). Mean trap catches were significantly higher for *An. funestus* s.l. than *An. gambiae* s.l. in Kerio Valley (χ^2^_1,86_ = 68.6, p < 0.0001) and Nguruman: (χ^2^_1,110_ = 102.7, p < 0.0001) (Fig. 2a).

### *Anopheles longipalpis* C dominates sibling species of *Anopheles funestus* identified

PCR to identify sibling species of *An. funestus* s.l. revealed variation in the species composition by area (Fig. 2b). In Kerio Valley, *An. longipalpis* C was predominant (75.7%, 362/478) followed by *An. funestus s.s.* (4.2%, 20/478) and *An. rivulorum* 0.8% (4/478). Species in this group were represented by *An. rivulorum* (26.4%, 19/72), *An. longipalpis* C (11.1%, 8/72) and *An. funestus* s.s. (2.8%, 2/72) in Rabai and in Nguruman by *An. rivulorum* (46.1%, 41/89) followed by 1.1% each of *An. longipalpis* C (1/89) and *An. funestus* s.s. (1/89). PCR amplification of DNA extracted from *An. funestus* s.l specimens was not possible for 28.3% (181/639) of the specimens collected, with Rabai (59.7%) having the highest number of unamplified samples (Fig. 2b). *Anopheles arabiensis* and *An. gambiae* were the two species observed in *An. gambiae* s.l. with the former occurring in higher proportion (41/46) than the latter (5/46) in Kerio Valley. Both species were not detected in samples collected from Rabai (Kilifi), but they were equally represented in the Nguruman samples (9/20 vs 11/20, *An. gambiae* and *An. arabiensis*, respectively).

### High *Plasmodium falciparum* sporozoite detected in *An. longipalpis* C and diverse cryptic species

Forty-six (46) of the 705 female anophelines tested positive for *P. falciparum* sporozoites (*Pf*sp) by high resolution melting (HRM-PCR) of the non-coding mitochondrial sequence (ncMS). A representative detection profile is shown in Fig. S1. This translated to an overall *Pf*sp rate of 6.5% but this finding varied with site and mosquito species (Table 1). In the *An. funestus* group, high *Pf*sp rates were recorded in *An. longipalpis* C (5.2%; 19/362) in Kerio Valley only; *An. funestus* s.s. in Kerio Valley (5%; 1/20) and Nguruman (100%; 1/1) and *An. rivulorum* in Nguruman (19.5%; 8/41) and Rabai (5.3%; 1/19). A significant proportion of unamplified cohorts also tested positive for *Pf*sp in Kerio Valley (12%;11/92) and Nguruman (4.3%; 2/46) (Table 1). *Pf*sp were detected in *An. gambiae* in Kerio Valley (20%; 1/5) and Nguruman (11.1%; 1/9), and in *An. arabiensis* from Kerio Valley (2.4%; 1/41).

**Table 1:** *Plasmodium falciparum* sporozoite rates (%) by species in selected dryland areas, Kenya.

	Species	Kerio Valley	Rabai	Nguruman	Total
*An. funestus* group	*An. longipalpis* C	5.2% (19/362)	0% (0/8)	0% (0/1)	5.1% (19/371)
	*An. funestus* s.s.	5% (1/20)	0% (0/2)	100% (1/1)	8.7% (2/23)
	*An. rivulorum*	0% (0/4)	5.3% (1/19)	19.5% (8/41)	14.1% (9/64)
	Unamplified	12% (11/92)	0% (0/43)	4.3% (2/46)	7.2% (13/181)
*An. gambiae* s.l.	*An. gambiae.*	20% (1/5)	0%	11.1% (1/9)	14.3% (2/14)
	*An. arabiensis*	2.4% (1/41)	0%	0% (0/11)	1.9% (1/52)
	Total	6.3% (33/524)	1.4% (1/72)	11% (12/109)	6.5% (46/705)

Number in parenthesis indicates proportion of the total that tested positive for each species.

**Figure 3:**
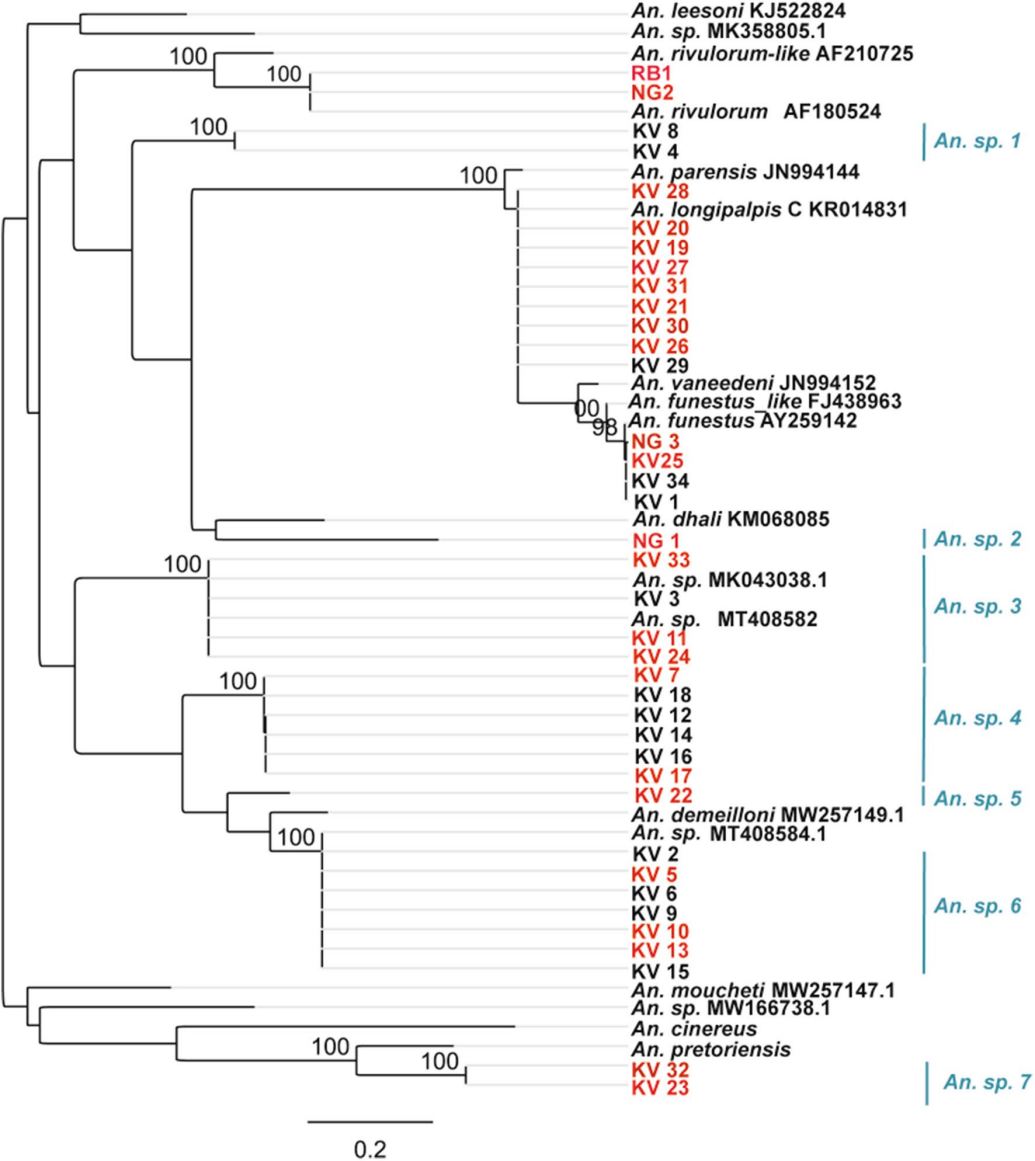
Phylogenetic tree for represetative mosquitoes morphologically scored as *An. funestus* group infected with *P. falciparum* sprozoites. Few samples negative for the malaria parasite are also included. Bootstrap support values are indicated above the nodes from 1000 replicates. Highlighted in red are the infected samples.

Further confirmation of the species identity of the infected specimens (for *An. funestus* group only) was conducted by PCR of the ITS2 region and then sequencing. Few samples that tested negative especially for the unamplified cohorts were included. Phylogenetic analysis of the generated sequences showed strong support (bootstrap (BS) = 98% and 89%) for *An. funestus* s.s. and *An. longipalpis* C, respectively (Fig. 3); two of the infected specimens clustered with *An. rivulorum* (BS = 100%; GenBank accession: AF180524). Additionally, sequenced samples of the unamplified cohort (positive and negative for *Pf*sp) resolved into well distinct clades (BS values = 100%) some clustering with previously reported but unidentified species viz: *Anopheles* sp.19 DZ 2020 (GenBank accession no: MT408584.1) and *Anopheles * sp. isolate A (GenBank accession no: MKO43038.1) indicating presence of cryptic species. Some of the positive specimens did not cluster with any referenced species (*An. *sp. 2, *An. *sp. 4, *An *sp. 5, *An. *sp. 7) (Fig. 3).

### Low levels of L119F-GSTe2 resistance alleles detected in *An. funestus* group and polymorphism analysis

**Figure 4:**
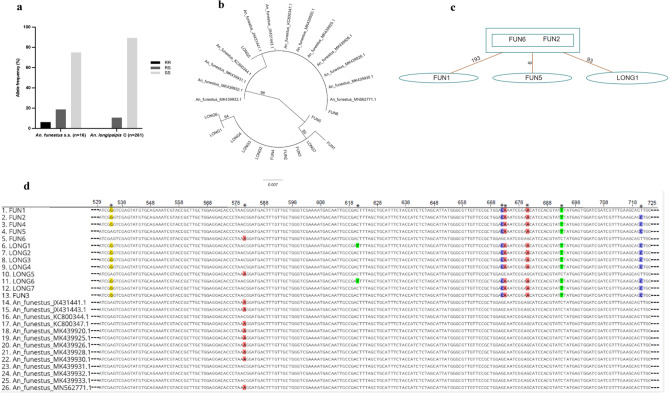
Analysis of the polymorphism of a portion of the glutathione S-transferase epsilon 2 (*GSTe2*) gene. (**a**) Allele frequency distribution, (**b**) ML phylogenetic tree of GSTe2 gene fragment with previously recorded haplotypes in *An. funestus* s.s. across Africa, (**c**) Templeton-Crandall Sing (TCS) haplotype network. The rectangles and ovals represent haplotypes with the lines linking haplotypes showing a single mutation event. (**d**) Aligned sequence traces showing the mutational differences/polymorphic positions in the haplotypes detected in Kenyan *An. longipalpis* C/*An. funestus* s.s.* indicates polymorphic positions.

The L119F-*GSTe2* mutation conferring metabolic resistance to DDT/pyrethroid^19^, was detected in *An. funestus* s.s*.* and *An. longipalpis* C. The frequency in the former species was 15.6% (1-RR, 3-RS, 12-SS) and in *An. longipalpis* C, 3.1% (0-RR, 28-RS, 233-SS) (Fig. 4a). Disaggregation of the data in *An. longipalpis* C by infection status found no difference in occurrence of the resistance allele (RS) between infected (12.5%) and non-infected (10.6%) cohorts (Fischer’s exact test, p = 0.68). A 240 bp fragment of the *GSTe2* gene was successfully sequenced in 17 mosquitoes, *An. longipalpis* C (7-RS and 3-SS) and *An. funestus* s.s. (6-RS and 1-RR). Phylogenetic analysis showed most haplotypes resolved as a distinct clade and few clustering with those previously detected in *An. funestus* s.s. from different parts of Africa (Fig. 4b). The haplotypes from Kenya exhibited genetic diversity with at least three mutational differences from those of *An. funestus* s.s. in GenBank (Fig. 4b–d) suggesting an independent occurrence, potentially from local selection.

## Discussion

Outdoor malaria transmission is a dynamic process and the vectors involved may vary according to the ecological setting. Here, we report diverse known and cryptic anopheline species collected outdoors and found to harbor *P. falciparum* sporozoites. These species could play critical roles in residual malaria transmission (RMT), given that parasite infection rate represents the most sensitive indicator in assessing importance of a vector species in this phenomenon^20^. Furthermore, we detected mutations in the *GSTe2* gene conferring resistance to pyrethroid/DDT in *An. funestus*/*An. longipalpis* C. This finding in a specific focus (i.e. Kerio Valley) potentially poses a risk to insecticide-based control measures and RMT^21^. This is the first report of the detection of this resistance marker in any species apart from *An. funestus* s.s. in the Funestus group. Overall, these findings may be useful in updating risk map of key vectors in Kenya^22^ and providing evidence-based decision in selection and implementation of appropriate interventions against malaria in the study areas.

We found that *An. funestus* s.l. was the predominant mosquito collected from the study areas. Molecular methods identified the sibling species *An. longipalpis* C, *An. funestus* s.s. and *An. rivulorum* in the Funestus group and other unidentified cryptic species, which varied with the site. Previous studies have largely implicated *An. funestus* s.s. as the most important vector in this group and known to highly adapt to dry season survival strategies^23^. Hence, we posed the question- could the low numbers signal dwindling importance of this species in certain ecological areas? Being a highly anthropophilic and endophilic mosquito^12,14,20^, the low numbers encountered outdoors is not surprising. It could also be argued that the low numbers reflect LLIN-related impact on their populations. The replacement of *An. funestus* s.s*.* by more outdoor resting/biting *An. rivulorum* after implementation of IRS in Kenya has been reported in the literature^24^. This could be the likely scenario following its absence in Rabai or enhanced contribution of *An. rivulorum* to malaria transmission in Nguruman and to a lesser extent in Rabai. Despite the low numbers, this species was still associated with parasite infection, which supports its high susceptibility and efficient role in malaria transmission. To assess its contribution to transmission in the study areas, longer term surveys, both indoors and outdoors, incorporating other trapping methods (e.g. human landing catches) and data on bed net coverage and use patterns in the target communities would be required.

Our findings revealed a high representation of lesser-known species, *An. longipalpis* C, in the Funestus group. The non-malaria vector status accorded *An. longipalpis* in southern Africa was attributed to absence of parasite infection and its highly zoophilic yet endophilic habits^25^. A high human blood meal index observed for this mosquito in Ethiopia^26^ lend support for its role as a malaria vector supported by previous detection of *P. falciparum* infection in indoor populations in Kerio Valley^12^. The present findings of high *Pf*sp rate and occurrence in high densities, even surpassing those of known malaria vectors, suggest it could assume a major role as a malaria vector in certain foci in Kenya. Understanding its biology and ecological adaption including breeding structure, resting and biting habits, and vectorial capacity are warranted.

Exophilic habits among mosquitoes do not preclude indoor activities and outdoor biting behavior could be a response to indoor insecticidal interventions^7^. The presence of mutations in the *GSTe2* gene conferring metabolic resistance to pyrethroids/DDT could be attributed to selection pressure from pyrethroid insecticide used in LLINs or use of agricultural insecticides. Endophilic behaviour is well documented in *An. funestus* s.s.^14,27^ and *An. longipalpis* C^12^. Increased vector survival/longevity has been observed as an important phenotype of L119F-*GSTe2* resistant *An. funestus* s.s.^28^. Thus, its detection could negatively impact vector control in this area through reduction in LLINs effectiveness^21^. Metabolic resistance produces an order of magnitude higher resistance to pyrethroids (commonly used on bednets) than that associated with *kdr* gene^29^. Furthermore, the mutational differences noted in the haplotypes of Kenyan *An. longipalpis* C/*An. funestus* s.s. compared to those of *An. funestus* s.s. elsewhere suggest an independent occurrence of resistance marker of the *GSTe2* gene. Further experimental work is needed to correlate the significance of the mutations on phenotypic response through insecticide exposure assays. Comparative transcriptomic and functional analyses are also needed to improve our understanding of the evolution of resistance in Kenyan populations of these species.

Our uncovering of diverse species in the Funestus group highlights the importance of application of molecular tools in routine entomologic surveillance^12,18^. Not only were diverse species uncovered, but they were also found associated with *P. falciparum* infection. One of the species in a well-supported clade (BS = 100%) had 100% sequence identity with *Anopheles* sp. 19 DZ-2020 (GenBank accession number MT408584.1) recently reported in western Kenya^17^ as a novel cryptic species. Another species shared 99.9% sequence identity with *Anopheles sp*. Isolate A (GenBank accession number MK043038.1) previously reported in the dryland area of West Pokot Kenya, as a potential secondary malaria vector on the basis of harboring malaria parasite and predominance among outdoor *An. funestus* mosquitoes^16^. Its vectoring ability was confirmed in a recent study in western Kenya^17^. The results suggest an adaption to dry ecological areas but highlight the potential for wider distribution and greater impact of these cryptic species on malaria transmission with growing effect of climate change and global warming. These could provide new, favorable and suitable environmental conditions for the survival of these species, but this needs further investigations. As control targets primary vector species, these species could fill the gap and emerge to become important vectors. Additionally, the observed number of unamplified specimens in the *An. funestus* group calls for improvement in the existing protocol to further identify and discriminate members of this group other than the commonly known malaria vectors.

In summary, this study has demonstrated the unexpected and role of *An. longipalpis* C in outdoor malaria transmission in a dryland ecology of Kenya. Furthermore, we detected mutations in the *GSTe2* gene conferring resistance to pyrethroid/DDT in *An. funestus* s.s*.*/*An. longipalpis* C in a specific focus (Kerio Valley) potentially posing a risk to insecticide-based control measures. The identified outdoor malaria vectors included known species (*An. funestus* s.s., *An. rivulorum*, *An. gambiae*, *An. arabiensis*), but at least six cryptic potential malaria parasite vectors most of which have not been previously described in Kenya. These species could play crucial roles in RMT, given that parasite presence represents the most sensitive indicator in assessing vector importance in this phenomenon^20^. Kenya is intensifying its national efforts in malaria control towards achieving elimination and success requires surveillance of all potential vector species. This could be facilitated by integration of molecular tools in routine entomologic surveillance^18^ to guide malaria control interventions. The outdoor biting and resting behaviour of these vectors underscores the need for complementary malaria control interventions since current conventional vector control tools in Kenya mainly target vectors that rest and bite indoors.

## Methodology

### Study sites, sample collection and preparation

Adult female mosquitoes used in this study had previously been collected from three areas: Kerio Valley (Baringo county), Rabai (Kilifi county) and Nguruman (Kajiado county) (Fig. 1), as part of vector-borne disease surveillance project and stored at – 80 °C at the International Centre of Insect Physiology and Ecology (*icipe*). The mosquitoes were surveyed between August 2019 and May 2020. Nguruman is an agropastoral area located in Kajiado county at the southern end of the Kenyan Rift Valley bordering Tanzania. The area has a semi-arid climate characterized by erratic rains, extreme temperatures, and cyclic and prolonged droughts^30^. The vegetation is dominated by bushland, grassland and open woodlands along seasonal river valleys. Specific indicator data for malaria is not available for Nguruman except for estimates pertaining to the larger Kajiado county which as of 2019 indicates a malaria incidence rate of 5 per 1000 population^31^. Collections in Kerio Valley (Baringo county within the Rift Valley) were conducted in Kapluk and Barwesa, both agro-pastoral areas with arid and semi-arid ecology. Malaria is a major vector-borne disease in the areas with report of perennially occurrence in neighboring riverine areas^32^. Rabai is one of the seven administrative sub-counties of Kilifi county in the coastal region of Kenya where malaria is endemic. The main economic activities in the area include subsistence agriculture, casual labor, crafts and petty trading. The weather patterns at the sites during the sampling period were as follows: Kerio Valley (mean daily temperature: 21.2 °C, mean daily rainfall: 4.1 mm, mean relative humidity: 73.4%); Rabai (mean daily temperature: 26.4 °C, mean daily rainfall: 2.1 mm; mean relative humidity: 78.1%) and Nguruman (mean daily temperature: 22.5 °C, mean daily rainfall: 0.9 mm, mean relative humidity: 61.2%).

### Mosquito survey and processing

Host seeking mosquitoes were trapped using CDC light traps baited with dry ice (carbon dioxide) attractive to several mosquitoes. Traps were set outdoors about 10–15 m away from randomly selected homesteads from 18:00 h to 06:00 h. After collection, the mosquitoes were anesthetized with trimethylamine and temporarily stored in liquid nitrogen before transportation to the Emerging Infectious Disease (EID) laboratory at *icipe* and later stored at − 80 °C. Anopheline mosquitoes were morphologically identified to species level using published taxonomic keys^15,33^.

### DNA extraction and *Anopheles* species discrimination

DNA was extracted from the head/thorax of individual mosquitoes using ISOLATE II Genomic DNA Extraction kit (Bioline, UK) following the manufacturer’s instructions and used for species discrimination and screening for *P. falciparum* infection and *Gste2* mutations (described below).

Cryptic sibling species of the *Anopheles funestus* and *Anopheles gambiae* complexes were identified using conventional PCR^34,35^ and/or sequencing. PCR for *An. funestus* complex in a 15 µl reaction volume comprised 0.5 µM of each primer targeting: *Anopheles funestus* s.s, *Anopheles vaneedeni*, *Anopheles rivulorum*, *Anopheles parensis*, *Anopheles leesoni*, *Anopheles longipalpis* A and *Anopheles longipalpis* C, 3 µl of 5X HOT FIREPol Blend Master Mix Ready to Load (Solis BioDyne, Estonia) and 2 µl of DNA template. The cycling conditions were initial denaturation at 95 °C for 15 min, and then 30 cycles of denaturation at 95 °C for 30 s, annealing at 46 °C for 30 s and extension at 72 °C for 40 s and final extension at 72 °C for 10 min. Size fragments of each species were scored after separation in 1.5% agarose gel electrophoresis stained with ethidium bromide against a 1 Kb DNA ladder (HyperLadder, Bioline, London, UK).

For *An. gambiae* s.l., PCR in a 10 µl volume consisted of 2 µl of 5X Evagreen HRM Master Mix (Solis BioDyne, Estonia), 1 µl of DNA template and 10 µM concentration of each primer targeting *An. gambiae* s.s and *An. arabiensis*. The thermal cycling conditions included initial denaturation for 15 min at 95 °C followed by 40 cycles of denaturation at 95 °C for 20 s, annealing at 61 °C for 15 s and extension at 72 °C for 20 s followed by final extension at 72 °C for 7 min.

A subset of *An. funestus* s.l. samples that failed to amplify using the established protocol, was further amplified and sequenced targeting the internal transcribed spacer 2 (ITS2) region of the ribosomal DNA (rDNA)^36^. This target has shown utility in discriminating closely related mosquito species including anophelines^12^ and sequences from diverse species for this marker are well represented in reference databases (e.g. GenBank). PCR volumes for rDNA ITS2 were 15 µl containing 0.5 µM of the forward and reverse primers, 3 µl of 5X HOT FIREPol Blend Master Mix Ready to Load (Solis BioDyne, Estonia) and 2 µl of DNA template. The cycling conditions were initial denaturation at 95 °C for 15 min, followed by 40 cycles of denaturation at 95 °C for 30 s, annealing at 60 °C for 30 s and extension at 72 °C for 45 s and final extension at 72 °C for 7 min. ExoSAP IT rapid cleanup kit (Affymetrix Inc., Santa Clara, CA, USA) was used to clean the PCR product as per the manufacturer’s guideline, and then outsourced for bidirectional Sanger sequencing to Macrogen, South Korea.

### Detection of malaria parasites

*Plasmodium falciparum* sporozoites in individual mosquitoes (head/thorax) were detected by analyzing high resolution melting (HRM) profiles generated from real time PCR products of non-coding mitochondrial sequence (ncMS)^37^. A *P. falciparum* DNA from National Institute for Biological Standards and Control (NIBSC; London, UK) was used as a reference positive control. PCR was carried out in a 10 µl volume consisting of 2 µl of 5X Evagreen HRM Master Mix (Solis BioDyne, Estonia), 1 µl of DNA template and 10 µM of each primer. PCR cycling conditions were initial denaturation for 15 min at 95 °C followed by 40 cycles of denaturation at 95 °C for 20 s, annealing at 61 °C for 15 s and extension at 72 °C for 20 s followed by final extension at 72 °C for 7 min. A fraction of RT-PCR-HRM positive samples were further analyzed using conventional PCR in a 10 µl volume consisting of 2 µl of 5X HOT FIREPol Blend Master Mix Ready to Load (Solis BioDyne, Estonia), 1 µl of DNA template and 10 µM of each primer. The cycling conditions comprised initial denaturation for 15 min at 95 °C followed by 40 cycles of denaturation at 95 °C for 20 s, annealing at 61 °C for 15 s and extension at 72 °C for 20 s followed by final extension at 72 °C for 7 min. PCR product of samples positive by RT-PCR were purified using ExoSAP- IT (USB Corporation, Cleveland, OH, USA) and outsourced for sequencing to Macrogen, South Korea. All sporozoite-positive mosquitoes were molecularly identified to species by PCR of the ITS2 region as described above.

### Genotyping for L119F-GSTe2 mutation and sequencing

Two outer and two inner primers in a PCR assay were used to genotype the L119F-GSTe2 mutations that confer resistance of *An. funestus* mosquitoes to pyrethroids/DDT^19^ as described previously^28^. Thus, only *An. funestus* s.l. was screened using this assay. Briefly, PCR in a 15 µl reaction volume consisted of 10 µM of each primer, 3 µl of 5X HOT FIREPol Blend Master Mix Ready to Load (Solis BioDyne, Estonia), and 2 µl of DNA template. The cycling conditions were initial denaturation at 95 °C for 15 min, followed by 40 cycles of denaturation at 95 °C for 30 s, annealing at 59 °C for 30 s and extension at 72 °C for 40 s and final extension at 72 °C for 7 min. Amplicons were resolved in a 1.5% agarose gel stained with ethidium bromide (Sigma-Aldrich, GmbH, Germany) against a 1 Kb DNA ladder (HyperLadder, Bioline, London, UK). The amplicons were scored as either homozygous susceptible (SS) at 312 bp, homozygous resistant (RR) at 523 bp or heterozygous (RS) when both bands were visualized.

Representative *GSTe2* allele positive samples were sequenced for the *GSTe2* gene using the Gste2F and Gste2R primers as described previously^38^. PCR comprised a reaction volume of 15 µl in MyTaq DNA Polymerase Kit (Bioline, London, UK) containing 10 µM of each primer, 5X My Taq reaction buffer, 2 µl of My taq DNA polymerase and 1 µl of DNA template. PCR conditions were: initial denaturation of 5 min at 95 °C, followed by 30 cycles of 94 °C for 30 s, 58 °C for 30 s and 72 °C for 1 min, with a final extension at 72 °C for 10 min. Cleaning and sequencing of amplicons were performed as described above.

### Sequence and polymorphism analysis

Sequences (mosquito*, P. falciparum*, *GSTe2*) were viewed and cleaned in Geneious Prime^39^ and queried in GenBank using Basic Local Alignment Search Tool (BLastn). Parasite sequences were assigned as *P. falciparum* after > 98% percentage identity. MAFFT in Geneious Prime^39^ was used to perform multiple sequence alignments with default parameters. Maximum likelihood (ML) trees were inferred for mosquito ITS2 sequences using the best fit model of sequence evolution with nodal support for different groupings evaluated through 1000 bootstrap replications. *GSTe2* gene polymorphism analysis was performed in Geneious Prime and ML tree reconstructed from MAFFT alignment using PhyML v. 2.2.4. Haplotype distribution network was constructed using Templeton-Crandall Sing (TCS) program v. 1.21^40^.

### Statistical analysis

Relative abundance was used to estimate the outdoor composition of the anopheline mosquitoes. Daily counts of female mosquito/trap/night for *An. funestus* s.l. and *An. gambiae* s.l. were compared for each area using generalized linear models (GLM) with negative binomial error structure based on best-fit model residuals. The mean catches/trap/night was computed for each of the species complexes. The *P. falciparum* sporozoite infection rates (*Pf*sp) were expressed as the number of positive specimens of the total number of specimens examined. The distribution of L119F-*GSTe*2 mutations was assessed by determining allelic frequencies in different species. Infection status among the resistant mosquitoes was compared using the Fisher’s Exact Test. Data were analyzed using R v 4.1.0 software at 95% confidence limit.

### Ethical considerations

Ethical review and approval of the study was granted by the Scientific and Ethical Review Unit (SERU) of the Kenya Medical Research Institute (KEMRI) (Protocol No. SSC 2787). Prior to data collection, the purpose of the study, procedures and associated benefits/risks were provided to the local leadership at county and community levels. Additionally, informed verbal consent to trap mosquitoes around homesteads was obtained from household heads.

## Supplementary Information

Supplementary Legends.

Supplementary Figure S1.

## Data Availability

All data supporting the conclusions of this article are included within the article. *Accession codes* Sequences of selected haplotypes of anopheline species harboring *Plasmodium falciparum* sporozoite infection and those without have been deposited in GenBank under accession numbers; *An. funestus* (OM857956-OMOM857959), *An. longipalpis* C (OM791454-OM791458), *An. rivulorum* (OM791451-OM791452) and (OM812265-OM812270 and ON146197-ON146198) unclassified *Anopheles* spp.
