# Supplementary material for: Outdoor malaria vector species profile in dryland ecosystems of Kenya
Source: Sci Rep. 2022 May 3;12:7131. doi: 10.1038/s41598-022-11333-2 (PMC9065082; doi:10.1038/s41598-022-11333-2)
Supplement: Supplementary file 1 — Supplementary Legends. [file 41598_2022_11333_MOESM1_ESM.docx]

**Supplementary Fig. S1**: Representative melt curves of *P. falciparum* infected samples. y-axis shows change in fluorescence units with increasing temperature (dF/dT), x-axis shows increasing temperature.
