# Supplementary figures and images for: Outdoor malaria vector species profile in dryland ecosystems of Kenya
Source: Sci Rep. 2022 May 3;12:7131. doi: 10.1038/s41598-022-11333-2 (PMC9065082; doi:10.1038/s41598-022-11333-2)

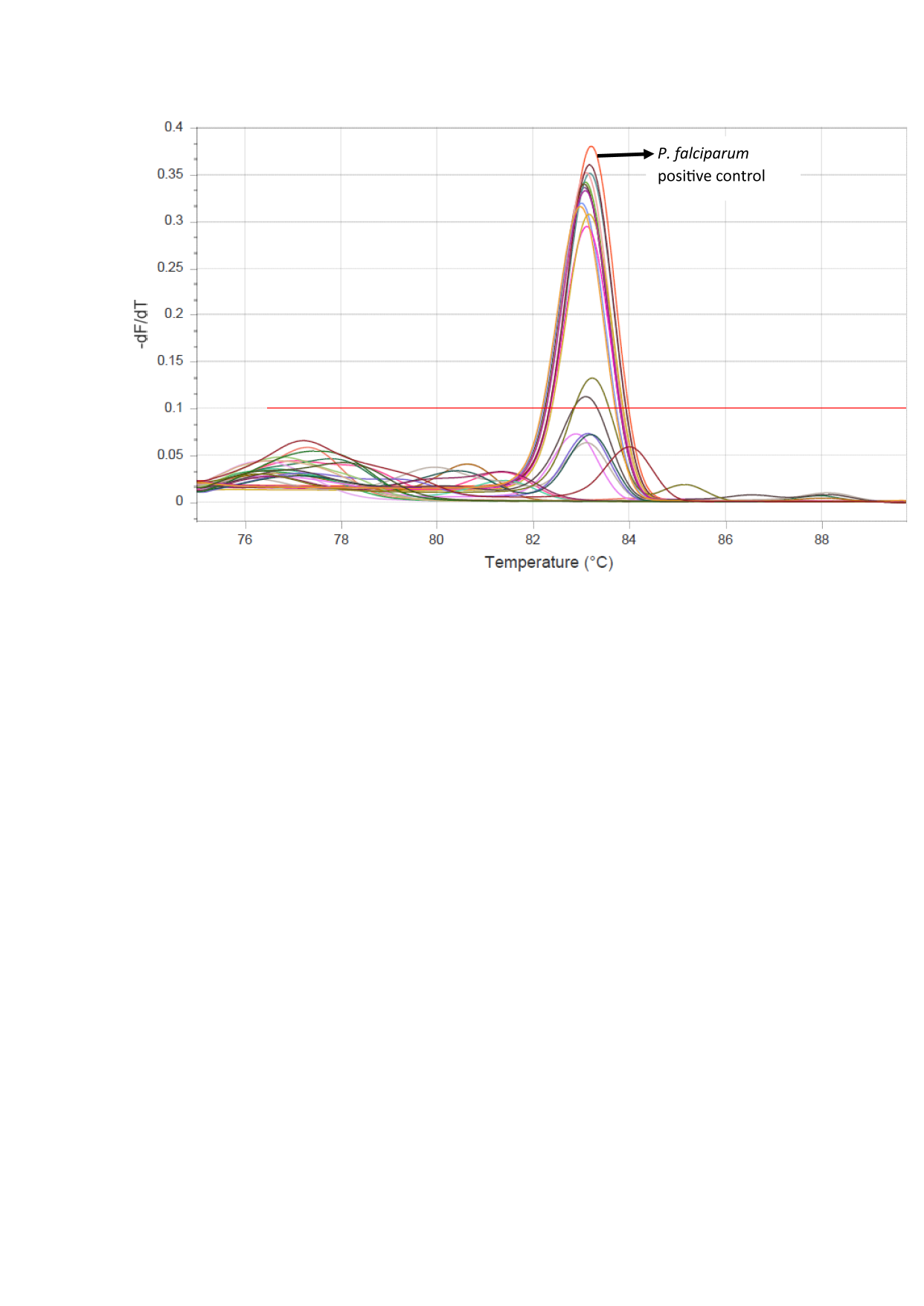

Supplement: Supplementary file 2 — Supplementary Figure S1. [file 41598_2022_11333_MOESM2_ESM.tif]
